# Leveraging COVID-19 Vaccine Safety Monitoring in Ethiopia and Pakistan to Enhance System-Wide Safety Surveillance

**DOI:** 10.9745/GHSP-D-23-00161

**Published:** 2024-02-20

**Authors:** Aida Arefayne Hagos, Zelalem Sahile, Waqas Ahmed, Souly Phanouvong

**Affiliations:** a Promoting the Quality of Medicines Plus, Addis Ababa, Ethiopia.; b Promoting the Quality of Medicines Plus, Islamabad, Pakistan.; c Promoting the Quality of Medicines Plus, Rockville, Maryland.

## Abstract

The rapid development, introduction, and global uptake of COVID-19 vaccines required countries to have strong pharmacovigilance systems in place to monitor and address adverse events following immunization (AEFIs). These systems provide timely data on vaccine safety that support decisions about the potential risks of vaccine adverse events relative to the benefit of disease prevention. In Ethiopia, the monitoring system was limited by the lack of data being submitted through its passive surveillance system, delays in investigating serious adverse events and conducting causality assessments, and the lack of reporting to the World Health Organization (WHO) global database, VigiBase. In Pakistan, the pharmacovigilance system lacked reporting requirements and guidance documentation, regulatory policies were insufficient, and staff lacked the capacity to evaluate AEFI reports. Several interventions were implemented in both countries to improve pharmacovigilance systems and processes necessary to collect, analyze, and report AEFIs from health care facilities to the national level and facilitate the use of global and national electronic reporting tools. In addition, Pakistan improved the regulatory policy environment and engaged vaccine manufacturers and private sector health facilities in AEFI reporting for the first time in the country. Outcomes include an increased number of COVID-19 vaccine-related AEFIs reported and causality assessments completed, which means that potential safety issues were being analyzed more quickly, comprehensively, and accurately. The number of AEFI reports submitted to VigiBase by Pakistan’s regulatory authority more than quintupled from approximately 5,000/quarter in 2021 to 28,555/quarter in 2022. In Ethiopia, by October 2022, 44,000 AEFI reports had been received, and 40 causality assessments completed. In both countries, timely AEFI data review and analysis led to prompt recommendations and regulatory actions, highlighting the far-reaching implications of strengthening the country-level pharmacovigilance systems. These strengthened systems are now in place for use with all vaccines.

## Abstract (precis)

This article demonstrates the importance of strengthening surveillance systems that monitor adverse events following immunization in Ethiopia and Pakistan to ensure vaccine safety and effectiveness and build public confidence in vaccination programs.

## Teaser

**Key Findings:**

Efforts in Ethiopia and Pakistan to strengthen existing pharmacovigilance systems for reporting adverse events following immunization (AEFIs) with the COVID-19 vaccine focused on:

- Developing robust pharmacovigilance tools for rapid response to ensure the swift collection, analysis, and reporting of post-vaccination safety data and enable quick responses to adverse events signals.
- Engaging stakeholders at all levels to adopt a collaborative approach to vaccine safety monitoring that facilitated efficient actions based on AEFI reporting data.
- Equipping staff with the necessary skills and tools to enable them to assess reports for not only COVID-19 vaccines but also other vaccines for comprehensive system-wide strengthening, enhancing the overall effectiveness of vaccine safety monitoring and reporting.

**Key Implications:**

- Policymakers, regulators, and donors should prioritize building surveillance and reporting systems, as well as engage and, where needed, build the capacity of different stakeholders (health workers, regulators, and manufacturers) to improve access to and decision-making on vaccine safety data.
- In an emergency setting, implementers should support the regulators and broader health system by mobilizing them to use the existing system for surveillance of medicinal products, including the introduction of any new products into existing systems.

Plain language article summary available.

## INTRODUCTION

The COVID-19 pandemic caused a significant loss of lives and economic impact worldwide. The rapid development and introduction of COVID-19 vaccines in 2021 raised public concerns about their safety, limiting their acceptance by some health care providers and the public. For all vaccines, particularly those for novel targets or that use unique technology, it is critical to have an effective pharmacovigilance system that has the ability to detect, collect, analyze, report, and make decisions about any adverse event following immunization (AEFI), defined as an “untoward medical occurrence which follows immunization and which does not necessarily have a causal relationship with the usage of the vaccine.”^1^

Such a system can ensure public safety, build and sustain public and provider confidence in the vaccines, and help ensure adequate uptake of potentially lifesaving measures that are critical to public health.^1^

The World Health Organization (WHO) estimates that “almost half of the world’s population lives in countries without an effective system for monitoring the safety of vaccines.”^1^ WHO recommends that all countries strengthen their ability to detect, investigate, assess, report, and respond to AEFIs before and during COVID-19 vaccine introduction.^2^

Vaccines undergo rigorous testing for safety and efficacy in clinical trials before they are approved for use in the population. Clinical trials study the vaccine in a relatively small number of study participants for a relatively short period of time compared to real-world use. This is particularly true when vaccines are put into use in an emergency (via emergency use authorization) before full regulatory approval.

As the vaccine is more widely used, it is important to monitor whether AEFIs are seen in larger, more heterogeneous populations over time. Vaccine safety monitoring systems are used to capture information on AEFIs in this larger population.^1^ According to WHO, AEFIs can range from minor common side effects, such as pain at the injection site, to more serious expected and unexpected events, known as serious adverse events (SAEs), including those requiring hospitalization or resulting in death.^3^ National regulatory authorities document AEFIs in individual case safety reports that they submit to VigiBase, the WHO Collaborating Centre for International Drug Monitoring global database of reported suspected adverse events of medicinal products, to identify possible risks to individuals from medicinal products, including vaccines.

National regulatory authorities seek to determine the likelihood that a vaccine caused an adverse event by conducting causality assessments. Ideally, these assessments at the national level are performed by a committee of reviewers with expertise in a range of medical disciplines (e.g., pediatrics, neurology, general medicine, forensic medicine, pathology, microbiology, immunology, and epidemiology).^1^ The committee should be free of external influences and should work closely with the national regulatory authority and the country’s immunization program. Causality assessments support decisions about what actions, if any, should be taken in response to adverse events. Such actions range from specifying conditions under which the vaccine should be administered to limiting the individuals to which it can be administered or to withdrawing the vaccine from the market.

In 2020, WHO published a safety surveillance manual for COVID-19 vaccines that recommended minimum requirements to ensure the safety of COVID-19 vaccines.^2^ Although the type and scope of vaccine safety monitoring activities that countries choose to implement depends on the resources available to them, WHO recommended that all countries aim to do the following:

WHO recommended that minimum requirements for safety surveillance of COVID-19 vaccines should include strengthened reporting and investigations of AEFIs.

- Strengthen routine passive surveillance reporting systems to handle the expected increase in frequency or severity (mild, moderate, and severe) of AEFIs.
- Detect and investigate safety signals or clustering of serious events, immunization errors, community concerns, etc.
- Perform systematic causality assessment of SAEs, clustered cases, and events above expected rate or of unusual severity.
- Prepare comprehensive plans to respond rapidly to any COVID-19 vaccine-related event, regardless of severity.
- Be able to respond to any concerns expressed by health care workers and maintain community confidence.^2^

Many entities have important roles to play in implementing these recommendations, including immunization programs, providers in public and private health facilities, vaccine manufacturers, facilities throughout the supply chain, vaccine safety committees, and national regulatory authorities. Each organization’s role and responsibilities must be clear, their efforts integrated, and information shared quickly. Staff must be qualified to fulfill their roles and have the tools and information systems to facilitate their work.

To strengthen AEFI surveillance systems to monitor the safety of COVID-19 vaccines in Ethiopia and Pakistan, the U.S. Agency for International Development (USAID) funded the Promoting the Quality of Medicines Plus (PQM+) program. This work was undertaken with COVID-19 American Rescue Plan funding from May 1, 2021 to September 30, 2022, in Pakistan and from October 1, 2021 to December 31, 2022, in Ethiopia. The PQM+ program helped to establish the institutional relationships and coordination mechanisms between health care facilities, regulators, and immunization programs required for effective pharmacovigilance; provided capacity-building for collecting, analyzing, and reporting AEFI data at facility, subnational, and national levels; and provided technical assistance and capacity-building in AEFI investigation and causality assessment. The program also supported the development of regulatory policies and guidelines and the use of electronic reporting tools for analyzing safety data and submitting AEFI data to WHO’s VigiBase.

In this article, we highlight the importance of strengthening pharmacovigilance systems, describe the interventions undertaken to strengthen COVID-19 vaccine safety surveillance systems in Ethiopia and Pakistan, and describe how the outcomes from these interventions can be leveraged to support system-wide safety surveillance for other medicinal products.

## PHARMACOVIGILANCE SYSTEM STRENGTHENING IN ETHIOPIA

Historically, Ethiopia used a passive system to monitor adverse events related to drugs and other medicinal products but did not regularly perform active surveillance (e.g., where authorities follow up on a cohort of vaccine recipients to monitor for health events). With a passive surveillance reporting system, health care providers and others (e.g., the public and manufacturers) voluntarily submit information on AEFIs and product defects (which might be caused by improper storage and handling of the vaccine). Reporting of AEFIs through the passive surveillance reporting system in Ethiopia was relatively limited. The number and types of COVID-19 vaccine AEFI reports collected and analyzed were small compared to the number of people vaccinated. Of almost 2.1 million COVID-19 vaccine doses administered as of July 2021, only 711 AEFI reports were received through the passive surveillance reporting systems. Furthermore, data from the National Pharmacovigilance Center showed there were delays in investigating whether reported SAEs were causally related to vaccine use. Before the PQM+ intervention, causality assessment was done for only 8 of the 36 reported SAEs following COVID-19 vaccination. In addition, Ethiopia’s reporting into WHO’s VigiBase was very limited. There was a need to strengthen the capacity of Ethiopia’s 12 regional AEFI task forces that investigate SAEs and the National Pharmacovigilance Advisory Committee that conducts causality assessments.

### Program Activities in Ethiopia

The PQM+ program worked with the Ethiopia Food and Drug Administration (EFDA) and its pharmacovigilance center to strengthen the pharmacovigilance system for vaccine safety surveillance. This included conducting an assessment of the AEFI monitoring system, providing training and technical support to the National Pharmacovigilance Advisory Committee on conducting investigations and causality assessments, and providing support to disseminate findings from active surveillance of 2 COVID-19 vaccines and submit AEFI reports to the WHO VigiBase. Staff from the 6 EFDA branch offices and regional health bureaus received training on the assessment tools and the data collection process.

#### Assessment of the AEFI Monitoring System

The PQM+ program and the USAID-funded Global Health Supply Chain-Procurement and Supply Management program provided technical and financial support to EFDA’s assessment of the AEFI monitoring system. The assessment identified gaps in the system, including lack of clarity on the AEFI reporting process; resource limitations to conduct timely investigation of SAEs; and training gaps at health facilities, Woreda health offices, regional health bureaus, and EFDA branch offices. The program recommended how to address these gaps. In June 2022, EFDA, with PQM+ program support, held a workshop with 65 data collectors and supervisors from EFDA branch offices and regional health bureaus to finalize data collection tools, provide training on the AEFI data collection process, and offer hands-on experience in assessing the AEFI monitoring system in administrative offices and health facilities.

#### Training on Causality Assessments

The PQM+ program helped build the capacity of Ethiopia’s Pharmacovigilance Advisory Committee to conduct a causality assessment of AEFIs from COVID-19 vaccines using WHO’s revised electronic causality assessment tool.^4^ In collaboration with WHO, the program trained the Advisory Committee, EFDA pharmacovigilance staff at headquarters and branch locations, staff from the decentralized pharmacovigilance centers at regional university hospitals, National Novel Oral Polio Vaccine task force members, and staff of the immunization program at the Ministry of Health on conducting a basic causality assessment. Subsequently, the Advisory Committee received advanced causality assessment training, enabling the use of the electronic causality assessment tool to perform assessments.

Ethiopia’s Pharmacovigilance Advisory Committee capacity was strengthened to conduct causality assessments of AEFIs from COVID-19 vaccines using WHO’s electronic assessment tool.

EFDA also received support from the program in investigating and documenting 20 SAEs following COVID-19 vaccinations at several hospitals in Addis Ababa to facilitate timely causality analysis. As a result, the Pharmacovigilance Advisory Committee made several recommendations to the National Immunization Program: (1) all vaccination sites should conduct a prescreening of vital signs before vaccination; (2) vaccine recipients should spend 15 minutes of observation time at the site following vaccination; (3) the adrenaline injection must be available at every vaccination site to manage allergic reactions and other SAEs; and (4) there should be financial support for proper diagnosis and treatment of individuals with SAEs, as this diagnosis is a crucial element of causality analysis. Implementation of these recommendations should enhance the handling of AEFIs and potentially reduce associated harm.

#### Improvement in Supply Chain Management of Vaccines and Passive Reporting

To prevent potential problems with vaccine quality (e.g., cold chains inadequate to comply with EFDA standards and requirements, inadequate qualification and calibration of equipment, and lack of temperature measuring devices), selected health workers at COVID-19 active surveillance sites and regional regulatory bodies were trained to detect, assess, and report COVID-19 vaccine and potentially related product defects. The PQM+ program provided training on basic principles of vaccine cold chain management at distribution sites and health facilities and cold chain requirements of the various types of COVID-19 vaccines available in Ethiopia. After the training, participants could identify potential quality issues that could arise from poor handling of vaccines in the supply chain, preparing them to detect and report any suspected quality defect. EFDA experts also demonstrated the reporting system and tools developed by the National Pharmacovigilance Center to prepare health care workers to use proper reporting channels.

#### Facilitation of Global Reporting to WHO Database

The PQM+ program facilitated the use of VigiFlow, a web-based reporting tool that streamlines the entry of adverse event reports into VigiBase^5^ and supported the National Pharmacovigilance Center with data entry from 2,100 COVID-19 vaccine AEFI reports into VigiBase.

#### Active Surveillance of COVID-19 Vaccines

The National Pharmacovigilance Center collected AEFI data using active surveillance for the Janssen (Johnson & Johnson) and Pfizer COVID-19 vaccines. The PQM+ program provided technical assistance to develop the protocol and train data collectors. Data were collected from approximately 10,000 vaccine recipients (for Janssen, individuals aged 18 years and older; for Pfizer, individuals aged 12 years and older) who were followed for 30 days. The data showed that the majority of AEFIs encountered by the participants were mild to moderate.

#### Dissemination of Safety Findings to Stakeholders

Results from the active surveillance and the national AEFI monitoring system were disseminated to 92 stakeholders from the Ministry of Health, National Immunization Program, regional health bureaus, EFDA branch offices, and other implementing partners. The dissemination event strengthened the engagement of major stakeholders and partners in vaccine safety monitoring and follow-up activities. It also helped build the foundation for immunization program implementers and regulatory bodies at various levels to collaborate on integrated advocacy work toward timely AEFI detection and reporting.

### Outcomes in Ethiopia

The EFDA, with support from the PQM+ program and USAID, improved its capacity to successfully perform passive and active surveillance of vaccines. Active surveillance was conducted on COVID-19 vaccines, and the results were disseminated. Regarding passive surveillance, capacity-building activities were conducted to sensitize health care providers at vaccination sites to report adverse events, including potential product quality defects. Those providers who were involved in assessing AEFIs were better prepared to perform their responsibilities, had increased the timeliness and quality of their efforts, and used the new WHO causality assessment tool. The evidence-based findings on the safety profile of the COVID-19 vaccines from these pharmacovigilance activities have been communicated with immunization program implementers and partners within Ethiopia.

As of June 2023, 44.9 million adults and children ages 12 years and older in Ethiopia were vaccinated against COVID-19. The immunization campaign was carried out in 4 rounds, and the most recent was delivered to adolescents in association with human papillomavirus routine National Immunization Program efforts. Integration of the immunization and national pharmacovigilance programs enabled the generation of safety data on COVID-19 vaccines. Through the PQM+ program’s efforts, during the fourth round of the immunization campaign, EFDA and Ethiopia’s Expanded Program on Immunization created a national radio spot to communicate the findings on the safety of COVID-19 vaccines in the Ethiopian population. This helped build public confidence and trust in vaccines and bolster routine immunization coverage.^6^^,^^7^

As of April 2023, the Pharmacovigilance Advisory Committee had performed causality assessments of 51 SAEs following COVID-19 vaccination, an increase from 40 causality assessments that had been completed by October 2022. The Advisory Committee also leveraged the training and continued to conduct causality assessments for 17 SAEs related to novel oral polio vaccine type 2, 4 related to the measles-containing vaccine, 1 for a nerve tissue-derived rabies vaccine, and 1 for a tetanus toxoid vaccine. Thus, the training led to immediate benefits related to other vaccines used by the Ethiopian health system.

Training on conducting causality assessments led to immediate benefits related to other vaccines used by the Ethiopian health system.

**FIGURE 1:**
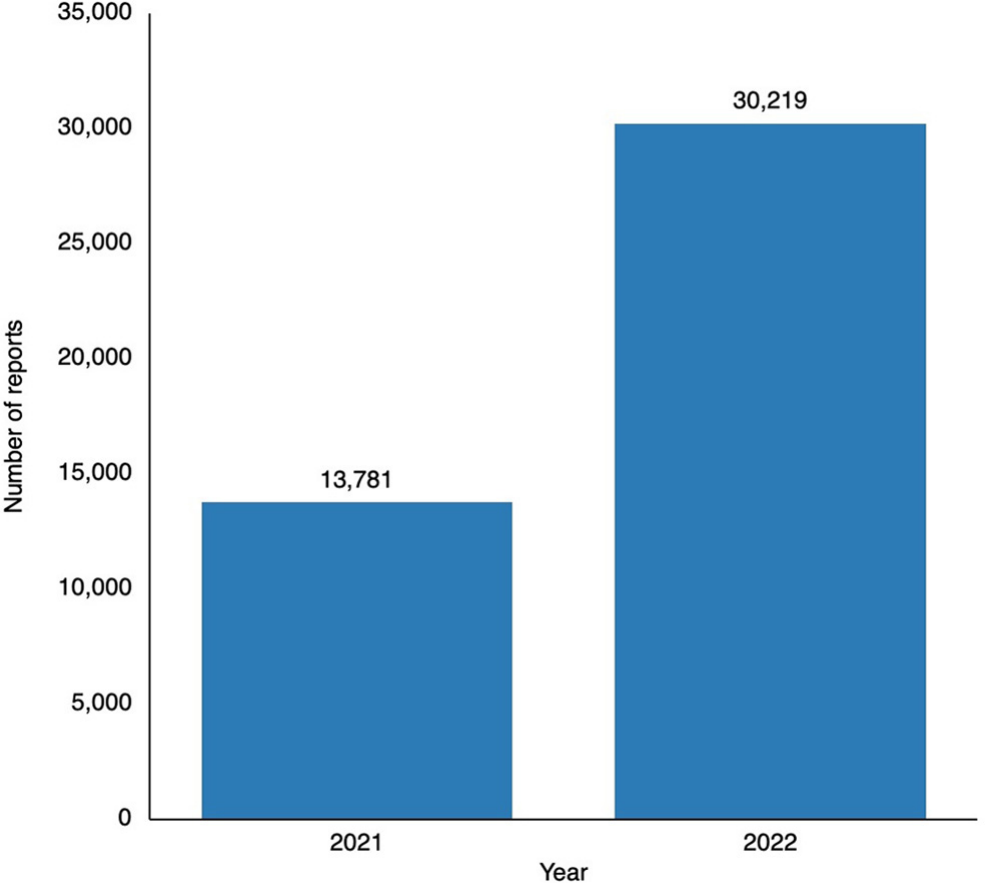
Reports of Adverse Events Following Immunization Captured in VigiFlow per Year in Ethiopia^a^ ^a^ Data source: Ethiopia Food and Drug Administration.

As a result of these interventions, 44,000 AEFI reports—primarily pertaining to COVID-19 vaccines—were submitted by health care professionals to the Ethiopian Pharmacovigilance Center between September 2021 and October 2022. Ethiopia had dramatically increased the submission of AEFI data into VigiFlow, with 21,640 reports by October 2022 to 30,219 by December 2022 (Figure 1). In October 2022, Ethiopia had the third-highest number of reports in VigiBase from an African country, with around 11,000 reports.

## PHARMACOVIGILANCE SYSTEM STRENGTHENING IN PAKISTAN

Using the WHO Global Benchmarking Tool for self-assessment,^8^ in 2019, the Drug Regulatory Authority of Pakistan (DRAP) identified pharmacovigilance to be its least-developed regulatory function. The assessment found that the system to report adverse drug reactions, AEFIs, or other safety data was not organized, and there was no requirement for reporting or guidance for the industry on how to submit vaccine safety data. Additionally, DRAP staff lacked the capacity to evaluate safety data and AEFI reports. A study of Pakistan’s pharmacovigilance system conducted in 2020 also found that the system was not fully functional.^9^^,^^10^

Pakistan’s regulatory policies regarding the authorization of COVID-19 vaccines and related pharmacovigilance were also identified to be insufficient. In April 2021, the Ministry of National Health Services, Regulations and Coordination (MONHSRC) developed but never adopted national guidelines for surveillance of COVID-19 vaccine AEFIs. These guidelines did not specify the roles/responsibilities of key stakeholders in the process, such as DRAP, Provincial Healthcare Commissions, and the Pakistan Expanded Program on Immunization. Of particular importance, the guidelines did not outline the role of health care providers in the private sector, where 70% of the Pakistani population obtains emergency care, including vaccinations.^11^

DRAP adopted the international practice of issuing Emergency Use Authorization (EUA) for COVID-19 vaccines to fight the pandemic. However, the requirements for safety and AEFI reporting by the vaccine manufacturers were not clearly mentioned in the EUA Certificates, as recommended according to international best practices.

Provincial AEFI committees that historically had supported childhood polio vaccination were already in existence; however, they struggled to operationalize their role in reviewing COVID-19 vaccine data and in regularly sharing data at the national level.

Additionally, manual entries of AEFI into VigiFlow were time consuming, and there was a relatively high risk of human transcriptional error in data entry. In light of these challenges, the PQM+ program implemented several activities to strengthen governance and reporting structures in Pakistan.

### Program Activities in Pakistan

#### Development of Guidelines and National Action Plan

The PQM+ program supported the revision and development of regulatory policies and guidelines for AEFI surveillance in Pakistan to ensure that all potential AEFIs related to COVID-19 vaccines were accurately reported. The National AEFI Guidelines for COVID-19 Vaccines and the National Action Plan for AEFI Surveillance of COVID-19 Vaccines were revised in consultation with Pakistan’s Federal Directorate of Immunization (FDI), WHO, and other federal and provincial stakeholders. Sixty-seven public and private sector stakeholders participated in a consultative session on the national guidelines held in October 2021. This session helped sensitize and integrate stakeholders who were previously not connected and had not participated in pharmacovigilance activities in Pakistan. The guidelines and action plan were approved by the MONHSRC and disseminated among the federal and provincial stakeholders for implementation. Although the documents developed were related to COVID-19 vaccines, they provide a framework that can be applied to other vaccines used in Pakistan’s childhood routine immunization program and emerging immunizations in older age groups.

With the support of the PQM+ program, COVID-19-related guidance documents were developed for (1) COVID-19 vaccine EUA holders on AEFI data collection, assessment, and reporting, as well as development and submission of risk management plans for COVID-19 vaccines; and (2) DRAP on risk-based post-marketing surveillance of COVID-19 vaccines. In addition, the EUA conditions were revised to engage EUA holders in AEFI reporting.

#### Revitalization of Provincial AEFI Committees

Provincial AEFI committees, which had formerly focused on childhood polio vaccines, were revitalized through the revision of their terms of reference and the inclusion of additional clinical experts on the committees. Standard operating procedures were developed for coordination among key stakeholders of the AEFI surveillance system, including the MONHSRC, FDI, DRAP, Provincial Healthcare Commissions, and health care facilities, as well as the COVID-19 vaccine manufacturer, importer, or distributor receiving EUA.

#### Establishing of Risk Assessment Expert Committee

At DRAP, the PQM+ program supported the establishment of a Pharmacovigilance Risk Assessment Expert Committee, which plays a critical role in EUA of products by evaluating adverse events that are not known or well known and quality issues detected by provincial and federal AEFI review committees on a priority basis. In addition, the committee recommends risk-minimization actions and communicates safety information on medicinal products to the public.

#### Development of Electronic Reporting Tools

With the revision of terms and responsibilities for EUA holders, the vaccine manufacturer, importer, or distributor became responsible for reporting known AEFIs associated with their products. The PQM+ program supported the development of an online portal where EUA holders could enter their AEFI data into the Pakistan Integrated Regulatory Information Management System, developed a user manual to assist EUA holders, and trained DRAP’s pharmacovigilance team to use the portal. The PQM+ program also developed an interface between the pharmacovigilance data in the information management system and VigiFlow. Previously, data were entered manually into VigiFlow, which slowed the reporting process. These electronic tools developed for COVID-19 vaccines can be used by all relevant stakeholders to report AEFIs for all vaccines used in Pakistan. It is worth noting that this tool can be used to report adverse reactions to not only vaccines but also any medicine in Pakistan.

Electronic tools developed for AEFI reporting for COVID-19 vaccines can be used to report AEFIs for all vaccines and adverse reactions to any medicine in Pakistan.

#### Capacity-Building

The PQM+ program undertook extensive capacity-building of:

- DRAP’s core pharmacovigilance staff on AEFI data collection and analysis.
- DRAP, provincial drug controllers, provincial quality control boards officials, and other government stakeholders from across Pakistan on risk-based post-marketing safety and quality surveillance of COVID-19 vaccines.
- The Provincial Healthcare Commissions and private health care providers on AEFI reporting for COVID-19 vaccines.
- DRAP and Provincial AEFI Investigation Committees on AEFI investigation and causality assessment.
- More than 200 stakeholders from health care commissions, the private sector, and EUA holders on AEFI reporting. This was the first training on AEFI reporting ever provided to private health care providers. Expert trainers were also trained to cascade the training to other stakeholders within the health system. Furthermore, these trainings can be applied beyond the COVID-19 vaccines because the same reporting channels are used for all vaccines.

### Outcomes in Pakistan

In Pakistan, AEFI reporting was not well established for routine or EUA vaccines before the use of COVID-19 vaccines. In the past, Pakistan compiled AEFI reporting only from childhood polio vaccines and only from public sector health facilities. Revision of the national guidelines on AEFI reporting equipped health authorities to engage the private sector in best practices for AEFI reporting. Importantly, engaging private sector health care providers and EUA holders in developing guidelines not only improved their knowledge and understanding of the process but also helped develop a sense of ownership and interest in further involvement in vaccination programs and pharmacovigilance. Bringing the private sector into AEFI reporting substantially increased the number of AEFI reports submitted to the system. The 3 sources of data on AEFIs now include public sector facilities and, for the first time, EUA holders and private sector facilities. The success of engaging the private sector in AEFI reporting for COVID-19 vaccines could serve as a model for other newly introduced vaccine programs in the future.

**FIGURE 2:**
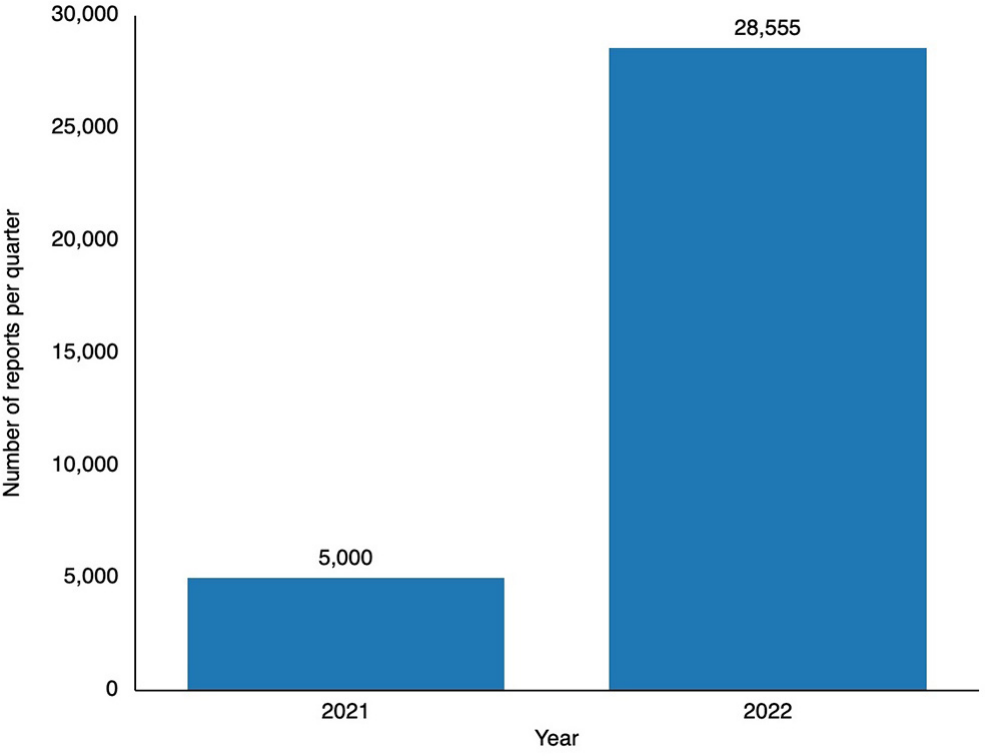
Reports of Adverse Events Following Immunization Captured in VigiFlow per Quarter in Pakistan^a^ ^a^ Data source: Drug Regulatory Authority of Pakistan.

The capacity-building activities improved the ability of health care workers to collect, analyze, report, and act on AEFIs related to COVID-19 vaccines, resulting in increased AEFI reporting rates and potential vaccine safety issues being promptly identified and addressed. With the development of the online portal and interface with the regulatory information management system, the pace of reporting increased substantially. As shown in Figure 2, the number of COVID-19 vaccine-related AEFI reports uploaded into VigiFlow by the regulatory authority of Pakistan more than quintupled from approximately 5,000/quarter at the start of the 2021 intervention to 28,555/quarter in 2022. Reporting from COVID-19 EUA holders increased from 0 to 12,001. All of these interventions helped greatly increase the Government of Pakistan’s visibility into vaccine safety.

Capacity-building activities improved the ability of health care workers to collect, analyze, report, and act on AEFIs related to COVID-19 vaccines, resulting in increased AEFI reporting rates.

The automated integration of AEFI data into VigiFlow also enhanced sharing of adverse event reports with WHO. This promotes global collaboration and empowers prompt global action when necessary, ensuring a coordinated response to vaccine safety issues.

Recently, the FDI, with WHO’s support, has included a surveillance component in its routine immunization tracking. This component will increase the surveillance and reporting capacity of health care providers involved in vaccination through FDI. Efforts made during the PQM+ intervention have had a lasting impact, with further improvements to the FDI system and integration of the immunization and AEFI reporting system.

In addition to improved reporting of AEFIs (in Pakistan and then into VigiBase), DRAP’s pharmacovigilance staff and Provincial AEFI Investigation Committees are better prepared to investigate AEFIs and conduct causality assessments for other vaccines and medicines. All the PQM+ interventions also contributed to building the public’s confidence on vaccination and leading the administration of at least 1 dose to the eligible population.^12^

## DISCUSSION

The COVID-19 pandemic highlighted the importance of vaccine safety monitoring and the need for strong AEFI surveillance systems. It caused immunization programs to shift from vaccines for children to ones that were prioritized for adolescents, adults, and elderly populations. The use of new vaccines that only had been approved for emergency use made effective pharmacovigilance even more important. Closer coordination among different parts of the health system, including the national immunization program, health service providers, vaccine manufacturers, and the regulatory authority, was and remains essential.

We describe the activities and results of system-based interventions to strengthen COVID-19 vaccine safety surveillance systems in Ethiopia and Pakistan, which have shown positive outcomes in strengthening pharmacovigilance systems in both countries.

Capacity-building activities on collecting, analyzing, reporting, and acting on vaccine safety data at various levels of the health system have enabled both countries to effectively monitor and address AEFIs related to COVID-19 vaccines and enabled monitoring of AEFIs in children as well as adolescents, adults, and the elderly. These activities have laid the foundation to expand to other vaccines, especially those introduced for routine use and have increased the number of AEFI investigations and SAE causality assessments completed, leading to prompt recommendations and actions to address vaccine safety issues.

Facilitating the use of electronic reporting tools, including at the facility level (in Ethiopia) and by EUA holders and private facilities (in Pakistan), enabled more comprehensive and timely AEFI reporting, data collection, and analysis. Increased reporting to VigiBase supports prompt global action when necessary.

The PQM+ program’s focus on assessing surveillance systems, strengthening AEFI guidelines, and promoting coordination among the many players responsible for pharmacovigilance in both the public and private sectors was instrumental in improving and institutionalizing pharmacovigilance in Ethiopia and Pakistan. In both countries, the changes in governance, processes, and regulatory systems established for COVID-19 vaccines are already being used for monitoring other vaccines.

In both countries, the changes in governance, processes, and regulatory systems established for COVID-19 vaccines are already being used for monitoring other vaccines.

### Challenges

Some challenges remain to be addressed in strengthening AEFI surveillance systems in these and other low- and middle-income countries.

More people working within the health system need to be adequately trained and informed about the importance of AEFI surveillance and how to collect, analyze, and report AEFIs effectively.^13^^,^^14^ SAE investigation is complex and time consuming; more people should be trained in conducting this important activity. Similarly, even with the use of the new WHO tool, causality assessment is challenging, so countries must develop and maintain this expertise.

As shown in this article, many diverse players at all levels of the health system in both the public and private sectors must be involved for pharmacovigilance to be effective. A system for developing standard operating procedures and ongoing orientation and training in concepts, procedures, and systems will be critical to address inevitable staff turnover.^13^

Another challenge is the lack of resources and infrastructure to support vaccine surveillance systems. Many low- and middle-income countries lack the necessary resources and infrastructure to conduct the investigations and to provide the training and information systems to monitor and address AEFIs effectively. Therefore, there is a need for increased investment in vaccine safety monitoring systems.

## CONCLUSION

This article has demonstrated the importance of strengthening AEFI surveillance systems in ensuring the safety and effectiveness of vaccines and building public confidence in vaccination programs. Capacity-building activities, facilitating the use of electronic reporting tools, and focusing on regulatory guidelines and governance were instrumental in improving AEFI surveillance in Ethiopia and Pakistan. This work also offers lessons for other countries on ways to strengthen pharmacovigilance and immunizations systems so that they are prepared for future public health emergencies.

One important legacy of the COVID-19 pandemic is improved linkages among all these parties and improved ongoing collaboration. A need remains for increased investment in vaccine safety monitoring systems including electronic information systems and training for health workers and regulators. Strengthening these systems will enable countries to monitor and address AEFIs related to COVID-19 vaccines, routine childhood immunization, and newly introduced vaccines, as well as the next public health emergency threat caused by a vaccine-preventable disease.

## Supplementary Material

GHSP-D-23-00161-supplement2.pdf

GHSP-D-23-00161-supplement3.pdf

GHSP-D-23-00161-supplement1.pdf

## Notes

**Peer Reviewed**

**First published online:** January 15, 2024.
