# Supplementary material for: Leveraging COVID-19 Vaccine Safety Monitoring in Ethiopia and Pakistan to Enhance System-Wide Safety Surveillance
Source: Glob Health Sci Pract. 2024 Feb 20;12(Suppl 1):e2300161. doi: 10.9745/GHSP-D-23-00161 (PMC10948120; doi:10.9745/GHSP-D-23-00161)
Supplement: GHSP-D-23-00161-supplement2.pdf [file GHSP-D-23-00161-supplement2.pdf]

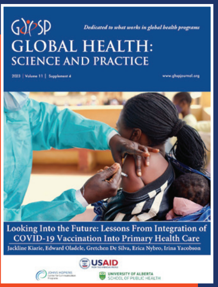

Tirer parti de la surveillance de la sécurité du vaccin contre la COVID-19 en Éthiopie et au Pakistan pour améliorer la surveillance de la sécurité à l'échelle du système : Résumé de l'article

Aida Arefayne Hagos, Zelalem Sahile, Waqas Ahmed and Souly Phanouvong

De quoi parle cet article ? Pour surveiller la sécurité du vaccin contre la COVID-19 au fur et à mesure de son utilisation dans une population, il est essentiel de disposer de systèmes de pharmacovigilance efficaces pour recueillir et analyser les données relatives à tous les effets secondaires suivant l'immunisation (ESSI). Cela contribue à garantir la sécurité continue des vaccins et de renforcer la confiance des gens dans les vaccins. En Éthiopie et au Pakistan, le programme de promotion de la qualité des médicaments Plus « Promoting the Quality of Medicines Plus », financé par l'USAID, a amélioré les systèmes de pharmacovigilance existants pour les vaccins afin de contrôler les données relatives à la sécurité des vaccins contre la COVID-19.

En Éthiopie, une évaluation du système de suivi des ESSI a révélé les lacunes.

- L'utilisation d'un système de surveillance passive a eu pour conséquence que le nombre et les types d'ESSI liés au vaccin contre la COVID-19 étaient faibles par rapport au nombre de personnes vaccinées.
- Il y a eu des retards dans l'enquête visant à déterminer si les ESSI signalés étaient causées par l'utilisation du vaccin.

Interventions :

- **Renforcer les capacités :** Formation du comité consultatif de pharmacovigilance de l'Éthiopie à la réalisation d'évaluations de causalité pour déterminer si les effets secondaires sont liés au vaccin contre la COVID-19.
- **Faciliter l'établissement de rapports mondiaux :** La saisie des données pour l'établissement de rapports sur les ESSI dans VigiFlow, un outil de

---

Les points de vue des auteurs

« Dans les deux pays, les changements en matière de gouvernance, de processus et de systèmes réglementaires mis en place pour les vaccins contre la COVID-19 sont déjà utilisés pour le suivi d'autres vaccins. »

- Aida Arefayne Hagos, conseiller technique, Promoting the Quality of Medicine Plus , Éthiopie

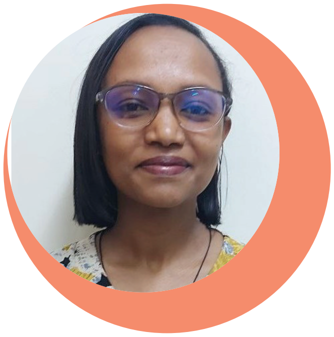

Lisez gratuitement l'article original publié dans Global Health Science and Practice à l'adresse suivante : https://doi.org/10.9745/GHSP-D-23-00161

rapports en ligne, a été rationalisée pour faciliter l'établissement de rapports mondiaux dans VigiBase, une base de données mondiale de l'Organisation mondiale de la santé (OMS).

- **Soutenir la surveillance active :** Un protocole de surveillance active a été élaboré et des collecteurs de données ont été formés pour suivre les personnes ayant reçu le vaccin et recueillir des données sur les ESSI. Les résultats ont été diffusés aux parties prenantes concernées.

Résultats :

- Augmentation du nombre d'évaluations de causalité effectuées par le comité consultatif de pharmacovigilance. Le comité est passé de 8 avant l'intervention à 40 d'ici octobre 2022. Des recommandations appropriées ont été formulées afin d'améliorer la prise en charge des ESSI et de réduire potentiellement les dommages associés.

- Augmentation spectaculaire de la transmission des données sur les ESSI dans VigiFlow et, par conséquent, dans VigiBase, l'Éthiopie se classant au troisième rang des pays africains ayant le plus grand nombre de rapports dans la base de données de l'OMS.

Au Pakistan, la pharmacovigilance a été identifiée comme la fonction réglementaire la moins développée du pays.

- Le système de surveillance des ESSI n'était pas entièrement fonctionnel et ne disposait pas de lignes directrices établies pour l'établissement de rapports sur les ESSI pour les vaccins de routine et les vaccins d'urgence.

- L'établissement de rapports sur les ESSI n'a été effectué que pour les vaccins antipoliomyélitiques destinés aux enfants et n'a pas pris en compte les vaccins du secteur privé, où 70 % de la population pakistanaise se fait vacciner et reçoit des soins d'urgence.

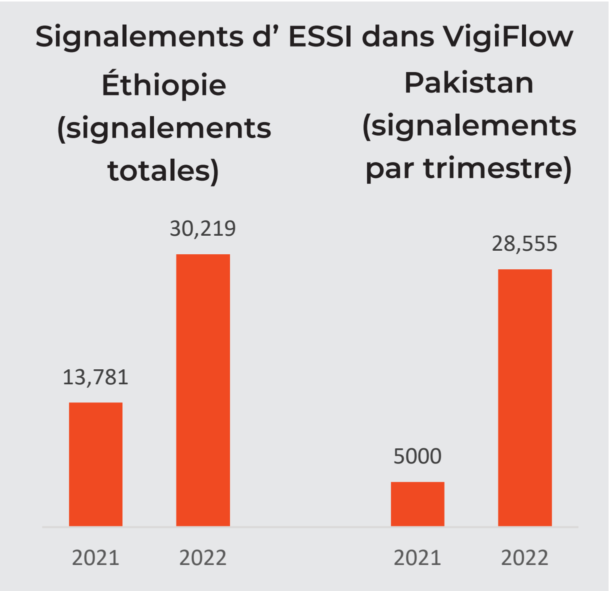

Interventions :

- **Renforcer les politiques et les lignes directrices réglementaires nationales :** Le gouvernement et d'autres parties prenantes ont collaboré à la mise à jour et à l'approbation des lignes directrices sur les ESSI des vaccins afin de s'assurer que les ESSI liés au vaccin contre la COVID-19 soient signalés.

- **Améliorer le suivi et le partage des données relatives au vaccin contre la COVID-19 par les moyens suivants :**
  - **Renforcer les rapports au niveau provincial** en élargissant les comités provinciaux sur les ESSI et

en leur donnant des normes de fonctionnement afin d'améliorer la coordination des données relatives au vaccin contre la COVID-19 entre les principales parties prenantes du système de surveillance des ESSI.

- **Faciliter et améliorer l'établissement de rapports électroniques** dans VigiFlow sur les ESSI concernant les vaccins contre la COVID-19.
- **Renforcer les capacités en matière d'établissement de rapports sur les ESSI, de collecte de données, d'analyse et d'évaluation de la causalité** pour les parties prenantes aux niveaux national et provincial, ainsi que pour les structures de soins de santé privés.

Résultats :

- **Augmentation du nombre de rapports sur les ESSI dans le système** provenant d'établissements des secteurs public et privé, ainsi que des autorisations d'utilisation en cas d'urgence.
- **Augmentation des rapports sur les ESSI liés aux vaccins contre la COVID-19 téléchargées dans VigiFlow** de 5 000 par trimestre au début 2021 à 28 555 par trimestre en 2022.

Que signifient ces résultats ? Le renforcement de la capacité nationale des systèmes de pharmacovigilance permet aux deux pays de surveiller et de traiter efficacement les ESSI liés aux vaccins contre la COVID-19 et à d'autres vaccins.

USAID FROM THE AMERICAN PEOPLE Knowledge SUCCESS

Ce guide a été réalisé grâce au soutien du peuple américain par l'intermédiaire de l'Agence américaine pour le développement international dans le cadre du projet Knowledge SUCCESS (Strengthening Use, Capacity, Collaboration, Exchange, Synthesis, and Sharing, accord de coopération n° 7200AA19CA00001 avec Johns Hopkins University. Knowledge SUCCESS est soutenu par le Bureau de la santé mondiale de l'USAID, le Bureau de la population et de la santé reproductive, et dirigé par le John Hopkins Centre for Communications Programs (CCP) en partenariat avec Amref Health Africa, le Centre d'économie comportementale de Busara et « FHI 360 ». Les informations fournies dans cette ressource relèvent de la seule responsabilité de Knowledge SUCCESS et ne reflètent pas nécessairement les opinions de l'USAID, du gouvernement américain ou de Johns Hopkins University.
