# Supplementary material for: Leveraging COVID-19 Vaccine Safety Monitoring in Ethiopia and Pakistan to Enhance System-Wide Safety Surveillance
Source: Glob Health Sci Pract. 2024 Feb 20;12(Suppl 1):e2300161. doi: 10.9745/GHSP-D-23-00161 (PMC10948120; doi:10.9745/GHSP-D-23-00161)
Supplement: GHSP-D-23-00161-supplement3.pdf [file GHSP-D-23-00161-supplement3.pdf]

Lições da integração da vacinação contra a COVID-19 nos cuidados de saúde primários | Série de resumos de artigos

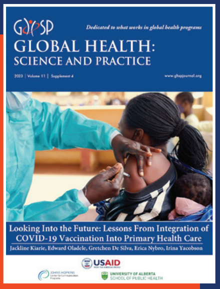

Aproveitar a monitorização da segurança da vacina contra a COVID-19 na Etiópia e no Paquistão para melhorar a vigilância de segurança em todo o sistema: resumo do artigo

Aida Arefayne Hagos, Zelalem Sahile, Waqas Ahmed and Souly Phanouvong

De que trata este artigo? Para monitorizar a segurança da vacina contra a COVID-19 à medida que esta é mais amplamente utilizada numa população, são essenciais sistemas de farmacovigilância eficazes para recolher e analisar dados sobre quaisquer eventos adversos após a imunização (EAAV). Isto ajuda a garantir a segurança contínua das vacinas e a reforçar a confiança das pessoas nas mesmas. Na Etiópia e no Paquistão, o programa Promoting the Quality of Medicines Plus, financiado pela USAID, melhorou os sistemas de farmacovigilância existentes para as vacinas, a fim de monitorizar os dados de segurança das vacinas contra a COVID-19.

Na Etiópia, uma avaliação do sistema de controlo do IEFA detetou lacunas.

- A utilização de um sistema de vigilância passiva resultou no facto de o número e os tipos de EAPV da vacina contra a COVID-19 serem reduzidos em relação ao número de pessoas vacinadas.
- Registaram-se atrasos na investigação para determinar se os EAPV notificados foram causados pela utilização da vacina.

Intervenções:

- **Reforçar capacidades:** o Comité Consultivo de Farmacovigilância da Etiópia recebeu formação sobre a realização de avaliações de causalidade para investigar se os eventos adversos estavam relacionados com a vacina contra a COVID-19.
- **Facilitar a comunicação a nível mundial:** a introdução de dados para a notificação de EAPV no VigiFlow, uma ferramenta de notificação

---

Perspetivas dos autores

"Em ambos os países, as mudanças na governação, nos processos e nos sistemas regulamentares estabelecidos para as vacinas contra a COVID-19 já estão a ser utilizadas para monitorizar outras vacinas."

- Aida Arefayne Hagos, consultora técnica, Promoting the Quality of Medicines Plus, Etiópia

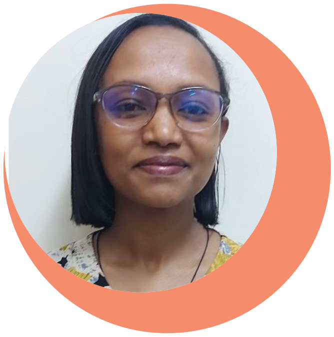

Leia o artigo original publicado na Global Health Science and Practice gratuitamente em:
https://doi.org/10.9745/GHSP-D-23-00161

baseada na Internet, foi simplificada para facilitar a notificação global no VigiBase, uma base de dados global da Organização Mundial de Saúde (OMS).

- **Apoiar a vigilância ativa:** foi desenvolvido um protocolo de vigilância ativa e os responsáveis pela recolha de dados receberam formação para acompanhar os indivíduos que receberam a vacina e recolher dados sobre os EAPV. Os resultados foram divulgados às partes interessadas relevantes.

Resultados:

- Aumento do número de avaliações do nexo de causalidade efetuadas pelo Comité Consultivo de Farmacovigilância realizadas, de 8 antes da intervenção para 40 até outubro de 2022. Foram apresentadas recomendações adequadas para melhorar o tratamento dos EAPV e reduzir potencialmente os danos associados.
- Aumento drástico da submissão de dados sobre os EAAV no VigiFlow e, por sua vez, para o VigiBase, com a Etiópia a ocupar o terceiro lugar entre os países africanos com o maior número de relatórios na base de dados da OMS.

No Paquistão, a farmacovigilância foi identificada como a função reguladora menos desenvolvida do país.

- O sistema de vigilância dos EAPV não estava totalmente operacional e não dispunha de diretrizes estabelecidas para a comunicação de EAPV relativos às vacinas de rotina e de emergência.

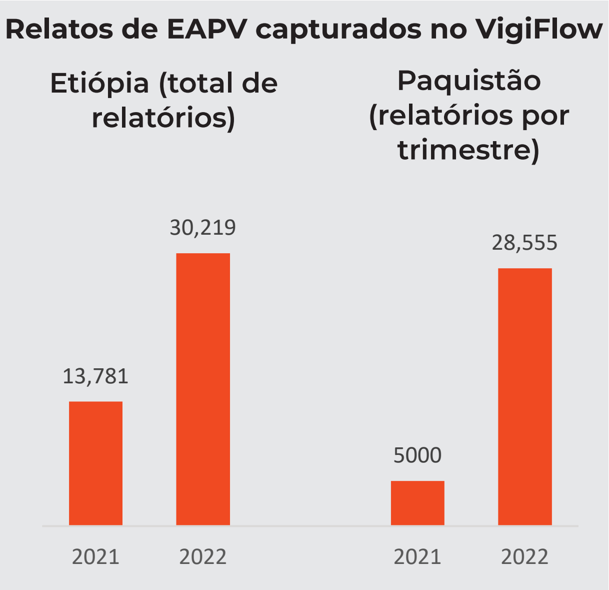

- A notificação de EAPV só foi feita para as vacinas contra a poliomielite infantil e não foi realizada para as vacinas do setor privado, onde 70% da população do Paquistão recebe vacinas e cuidados de emergência.

Intervenções:

- **Reforçar as políticas e diretrizes regulamentares nacionais:** o governo e outras partes interessadas colaboraram na atualização e aprovação de diretrizes sobre EAPV para garantir que os que são relacionados com a vacina contra a COVID-19 fossem comunicados.
- **Melhorar a monitorização e o compartilhamento de dados sobre as vacinas contra a COVID-19 através de:**
  - **Reforço dos relatórios a nível provincial** através do alargamento dos comités provinciais de EAPV e da definição de normas de funcionamento para melhorar a coordenação dos dados sobre a vacina contra a COVID-19 entre as principais partes interessadas no sistema de vigilância dos EAPV

- **Facilitação e melhoria da comunicação eletrónica** no VigiFlow dos EAPV relacionados com as vacinas contra a COVID-19
- **Reforço da capacidade de comunicação, recolha de dados, análise e avaliação da causalidade** dos EAPV para as partes interessadas a nível nacional e provincial, bem como para os estabelecimentos de saúde privados.

Resultados:

- **Aumento do número de relatórios de EAPV no sistema**, provenientes de unidades dos setores público e privado, bem como de autorizações de utilização de emergência.
- **Aumento dos relatórios de EAPV relacionados com a vacina contra a COVID-19 carregados no VigiFlow** de 5.000 por trimestre no início de 2021 para 28.555 por trimestre em 2022.

O que significam estes resultados?

O reforço da capacidade nacional dos sistemas de farmacovigilância permite que ambos os países monitorizem e tratem eficazmente os EAPV relacionados com as vacinas contra a COVID-19 e outras vacinas.

USAID FROM THE AMERICAN PEOPLE

Knowledge SUCCESS

Este guia resumido é possível graças ao apoio do povo americano através da U.S. Agency for International Development ao abrigo do Acordo de Cooperação do Projeto Knowledge SUCCESS (Strengthening Use, Capacity, Collaboration, Exchange, Synthesis, and Sharing) n.º 7200AA19CA00001 com a Universidade Johns Hopkins. O Knowledge SUCCESS é apoiado pelo Gabinete de Saúde Global, da População e Saúde Reprodutiva da USAID e dirigido pelo Johns Hopkins Center for Communication Programs (CCP) em parceria com a Amref Health Africa, The Busara Center for Behavioral Economics (Busara) e a FHI 360. As informações fornecidas neste guia resumido são da exclusiva responsabilidade da Knowledge SUCCESS e não refletem necessariamente as opiniões da USAID, do Governo dos EUA ou da Universidade Johns Hopkins.
