# Supplementary material for: Leveraging COVID-19 Vaccine Safety Monitoring in Ethiopia and Pakistan to Enhance System-Wide Safety Surveillance
Source: Glob Health Sci Pract. 2024 Feb 20;12(Suppl 1):e2300161. doi: 10.9745/GHSP-D-23-00161 (PMC10948120; doi:10.9745/GHSP-D-23-00161)
Supplement: GHSP-D-23-00161-supplement1.pdf [file GHSP-D-23-00161-supplement1.pdf]

Lessons from Integration of COVID-19 Vaccination Into Primary Health Care | Article Summary Series

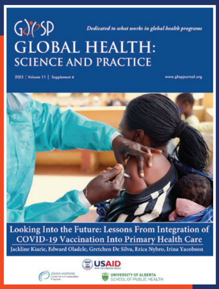

Leveraging COVID-19 Vaccine Safety Monitoring in Ethiopia and Pakistan to Enhance System-Wide Safety Surveillance: Article Summary

Aida Arefayne Hagos, Zelalem Sahile, Waqas Ahmed and Souly Phanouvong

What is this article about? To monitor the safety of the COVID-19 vaccine as it is being more widely used in a population, effective pharmacovigilance systems are critical for collecting and analyzing data on any adverse events following immunization (AEFIs). This helps assure the ongoing safety of the vaccines and build people's trust in the vaccines. In Ethiopia and Pakistan, the USAID-funded Promoting the Quality of Medicines Plus program improved the existing pharmacovigilance systems for vaccines to monitor safety data for COVID-19 vaccines.

In Ethiopia, an assessment of the AEFI monitoring system found gaps.

- The use of a passive surveillance system resulted in the number and types of COVID-19 vaccine AEFI reports being small compared to the number of people vaccinated.
- There were delays in investigating whether the reported AEFIs were caused by vaccine use.

Interventions:

- **Build capacity:** Trained Ethiopia's Pharmacovigilance Advisory Committee on conducting causality assessments to investigate whether adverse events were related to the COVID-19 vaccine.
- **Facilitate global reporting:** Data entry for AEFI reporting to VigiFlow, a web-based reporting tool, was streamlined to make it easier for global reporting into VigiBase, a World Health Organization (WHO) global database.
- **Support active surveillance:** An active surveillance protocol was developed, and data collectors were trained to follow up individuals who received the vaccine and collect data on AEFIs. Results were disseminated to relevant stakeholders

Results:

- Increased the number of causality assessments that the Pharmacovigilance Advisory Committee performed from 8 before the intervention to 40 by October

Insights from the Authors

"In both countries, the changes in governance, processes, and regulatory systems established for COVID-19 vaccines are already being used for monitoring other vaccines."

- Aida Arefayne Hagos, Technical Advisor, Promoting the Quality of Medicine Plus, Ethiopia

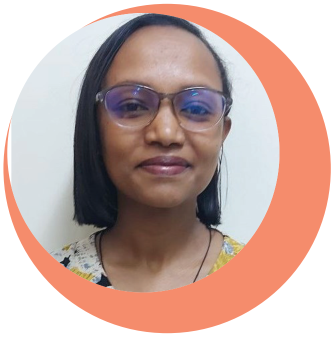

Read the original article published in Global Health Science and Practice for free at:
https://doi.org/10.9745/GHSP-D-23-00161

2022. Appropriate recommendations were provided to enhance handling of AEFIs and potentially reduce associated harm.

- Increased AEFI data submission into VigiFlow dramatically and, in turn, into VigiBase, with Ethiopia ranking third among African countries in having the highest number of reports in the WHO database.

In Pakistan, pharmacovigilance was identified as Pakistan's least-developed regulatory function.

- The system for AEFI surveillance was not fully functional and lacked established guidelines for reporting AEFIs for routine and emergency use vaccines.
- AEFI reporting was only done for childhood polio vaccines and missing those from the private sector, where 70% of Pakistan's population obtain vaccinations and emergency care.

Interventions:

- **Strengthen national regulatory policies and guidelines:** Government and other stakeholders collaborated to update and approve AEFI guidelines to ensure AEFIs related to the COVID-19 vaccine were reported.
- **Improve COVID-19 vaccine data monitoring and sharing by:**
  - **Strengthening provincial-level** reporting by expanding provincial AEFI committees and giving them operating standards to improve coordination of COVID-19 vaccine data among key stakeholders in the AEFI surveillance system

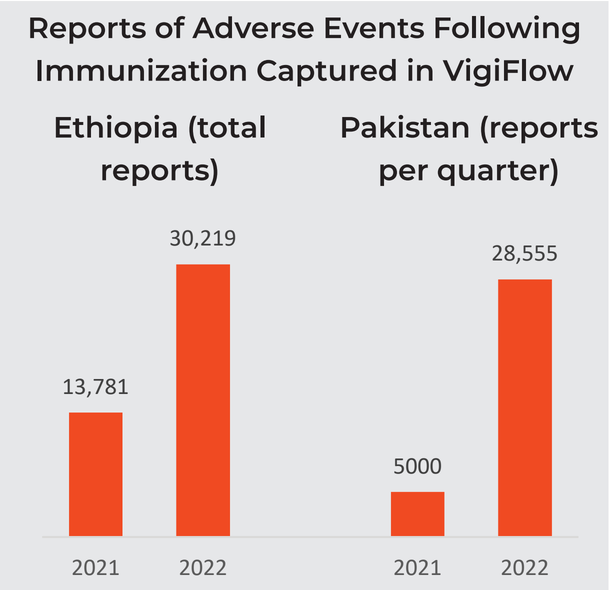

  - **Facilitating and improving electronic reporting** into VigiFlow of AEFIs on COVID-19 vaccines
  - **Building capacity on AEFI reporting, data collection, analysis, and causality assessment** for national- and provincial-level stakeholders, as well as private health care facilities.

Results:

- **Increased the number of AEFI reports** into the system from facilities in the public and private sectors, as well as emergency use authorization.
- **Increased COVID-19 vaccine-related AEFI reports uploaded into VigiFlow** from 5,000 per quarter in early 2021 to 28,555 per quarter in 2022.

What do these results mean?

Strengthening the national capacity of pharmacovigilance systems enables both countries to effectively monitor and address AEFIs related to the COVID-19 vaccines and other vaccines.

This summary brief is made possible by the support of the American People through the U.S. Agency for International Development under the Knowledge SUCCESS (Strengthening Use, Capacity, Collaboration, Exchange, Synthesis, and Sharing) Project Cooperative Agreement No. 7200AA19CA00001 with the Johns Hopkins University.
Knowledge SUCCESS is supported by USAID's Bureau for Global Health, Office of Population and Reproductive Health and led by the Johns Hopkins Center for Communication Programs (CCP) in partnership with Amref Health Africa, The Busara Center for Behavioral Economics (Busara), and FHI 360. The information provided in this summary brief are the sole responsibility of Knowledge SUCCESS and does not necessarily reflect the views of USAID, the U.S. Government, or the Johns Hopkins University.
